# M. Duclos on Fissure of the Anus in Infants

**Published:** 1846-08

**Authors:** 


*M. Duclos on Fissure of the Anus in Infants.*—It is a generally received opinion, says M. Duclos, that fissure of the anus is not a disease to which infants are subject. M. Trousseau's experiments, however, at the Neckar Hospital, proves that such is not the case, and that infants, even during suckling, are liable to this distressing disease, as well as adults. M. Duclos gives, in the *Journal de Chirurgie*, two cases, which occurred under M. Trousseau.

The first case is that of a little girl, one year old, under treatment for a white swelling of the knee. This child had been constipated from her birth, but more especially for the four previous months, the bowels being moved only every third or fourth day. Two months previous, the mother remarked that every time the bowels were opened the child screamed violently. The pain appeared to commence with the effort of defecation, to continue during the passage of the fæcal matter, and to be prolonged for a few seconds afterwards. For the last month, more especially, defecation had been exceedingly painful, and at each stool the child had voided a few drops of blood, either before or after the fæces, but never mixed with them. Sometimes the child, after a violent effort, would void a few drops of pure blood, scream violently, and make an effort as if to prevent the escape of fæces, in which case no stool took place. The general health was very good.

On examining the anus, the following was found to be the state of the parts:—The circumference of the anus was perfectly healthy, but on deeply separating the folds, at the anterior part and between two folds of skin, a fissure, a millimetre in width and about five millimetres in length, of a red colour, was distinctly perceived. It was the more clearly seen, as the child, screaming violently, protruded the anus. The constriction at the anus was so great that the extremity of the little finger could scarcely be introduced. M. Trousseau prescribed an enema, composed of extract of rhatany, one scruple; water, three ounces. The child kept the injection for five minutes, and returned it along with soft fæces. The injection was repeated daily for five days. Each time the passage of the fæces appeared less painful, and on the sixth day the injection was discontinued. The motions were then easy, free from blood or pus, and unaccompanied by pain. The child left the hospital ten days after, quite cured of the anal affection.

The second case occurred in M. Trousseau's private practice. The child, eight months of age, well developed, and in previous good health, had been suckled by the mother until the age of six months and a half. At that epoch, it was weaned, and was subsequently attacked with violent diarrhœa, which gave way under the use of emollients. The diarrhœa was followed by obstinate constipation. This state had existed for about eight days, when the child was seized, during defecation, by violent pain at the anus, and the fæces were found tinged with blood. From that time, the child suffered great pain on defecation, and for some minutes afterwards. The fæces were hard, and generally tinged with a few drops of blood. The child was constipated. General state satisfactory. On examining the anus, around its orifice there were found a little erythema and eczema, which had been occasioned by the diarrhœa, and were fast dying away. Behind, and to the left, on separating the folds of the anus, a fissure, about two millimetres in length and one in depth, of a rosy colour, was discovered. It was very distinctly seen on the child's protruding the anus in an effort for defecation. The anus was considerably constricted.

The same treatment was adopted as in the former case, and with equal success. The fissure cicatrized completely in about ten days, all pain on the evacuation of the fæces disappearing.

In these cases, says M. Duclos, the fissures were perceptible to the eye; but the seat of the pain, the mode of its manifestation, and the slight hæmorrhage on defecation, could leave but little doubt in the mind of the observer as to the nature of the disease, even in the absence of ocular demonstration. The symptoms, however, do not, if we judge from these cases, present absolutely the same form as in adults. In adults, the pain is generally most severe during the first hours which follow the excretion of the fæcal matters. Often, indeed, it gradually increases for some time after the motion, until it attains an intolerable intensity. With the children whose cases are reported above, nothing similar occurred. The pain seemed to cease a few minutes after the escape of the fæces, only to reappear on the bowels being again moved. Nothing appeared to indicate the presence of the slightest pain after the excretion of the fæcal matter. It is difficult to account for the fact.

Another circumstance is worthy of notice—viz., the slight hæmorrhage which invariably accompanied each alvine evacuation. In adults, extensive and very painful fissures may exist, without there being the slightest loss of blood. M. Velpeau appears even to think that it is the general rule.

The rapidity with which these fissures were cured by the rhatany injections is worthy of notice. Is a rapid cure to be expected in all such cases with young infants under similar treatment?—*London Lancet.*

